# The strategies of exercise intervention for adolescent depression: A meta-analysis of randomized controlled trials

**DOI:** 10.3389/fpsyg.2022.974382

**Published:** 2023-01-04

**Authors:** Chang Sheng Zhang, Liang Cheng, Xiaoan Chen, Yi Wang, Shuguang Wei, Jinxiu Sun

**Affiliations:** ^1^School of Physical Education, Chengdu Sport University, Chengdu, China; ^2^School of Sports Medicine and Health, Chengdu Sport University, Chengdu, China; ^3^School of Sports Science, Jishou University, Jishou, Hunan, China; ^4^Department of Psychology, College of Education, Hebei Normal University, Hebei, China

**Keywords:** adolescent, depression, exercise intervention, exercise strategies, meta-analysis

## Abstract

**Purpose:**

This study aimed to investigate the effect of exercise intervention, and analyze exercise intervention strategies for adolescent depression through a meta-analysis of RCTs.

**Methods:**

Accordance to PRISMA guidelines, PubMed, Medline, EBSCO, Web of Science, SPORTDiscus, PsycINFO, ProQuest, and CNKI were searched for eligible records. Peer-reviewed studies were included if they met the following criteria: population (mean age of 10–18 years), intervention (physical activity, sport, or exercise), and outcomes (depression, adherence, ITT, dropout, adverse events, follow-up report). The protocol of this systematic review was registered in PROSPERO (CRD42022321683). Effect sizes calculations and methodological quality of exercise intervention (TESTEX scale) were carried out. The certainty of evidence was assessed by GRADE framework.

**Results:**

Thirteen randomized controlled trials were eligible for this review, which comprised a total of 433 adolescents. Compared with the control treatment, the effect of exercise on adolescent depression was moderate (SMD = −0.65, 95%CI: −1.03 to −0.27, *p* < 0.01). Heterogeneity was substantial (*T*^2^ = 0.30, *I*^2^ = 67%, *p* < 0.01). The moderating effect analysis showed that exercise intervention characteristics (organization form, exercise frequency, exercise intensity, exercise type, and single exercise session duration) of included studies varied greatly revealing multiple factors that may impact the antidepressant effect of exercise on adolescent depression (*I*^2^ > 50%, *p* < 0.05). Three studies show that the positive effect of exercise on reducing depression in adolescents remained 40 weeks after the intervention. Moreover, owing to the included studies contained methodological limitations, the certainty of evidence was reduced to moderate level.

**Conclusion:**

This study shows that exercise intervention has a moderate and sustained positive effect on adolescent depression. Our results recommended that adolescents with depression undertake moderate to high intensity group mixed exercise for more than 12 weeks, 20 to 60 min/time, more than 3 times/week. Additionally, our study also shows that the antidepressant effects remained for a long time after the end of exercise interventions. However, following the GRADE framework, we rated the certainty of evidence the primary meta-analysis as moderate evidence due to some limitations of included studies. Therefore, rigorous studies are still needed to verify the results.

**Systematic review registration:**

[https://www.crd.york.ac.uk/PROSPERO/display_record.php?RecordID=321683], identifier [CRD42022321683].

## Introduction

Depressive symptoms and depressive disorders are a common threat to the mental health of adolescents (World Health Organization, 2020). Depressive disorder is diagnosed when depressive symptoms are present for most days over at least 1 year (Thapar et al., 2012). According to the Centers for Disease Control and Prevention, an estimated 4.4 million adolescents in the United States had been diagnosed with depression as of 2016 (Centers for Disease Control and Prevention of American, 2018). According to the latest Chinese official data, the overall prevalence of depression among Chinese teenagers is 15.4% (Huang et al., 2019). However, it should be noted that the official or relevant statistical data are only obtained through clinical diagnosis or self-rating depression scale screening. In reality, there are still a considerable number of children and adolescents with depression due to the sense of medical fraud (Dunn et al., 2005), concealment of disease (Costello et al., 2006), and other factors are not detected. This situation means that a significant number of adolescents with depression do not receive timely intervention. What is more important is that the negative effects of depression can extend to the employment and social status of teenagers, and even lead to self-harm or suicide in serious cases (Clayborne et al., 2019). Depression of adolescent imposes a heavy burden of disease on countries, societies and individuals (Kessler and Bromet, 2013; Sagatun et al., 2016). Therefore, this study will focus on the active prevention and treatment of depression and depressive symptoms in adolescents.

At present, the treatment of adolescent depression mainly includes drug therapy and psychological therapy (Weisz et al., 2017). In drug therapy, clinical guidelines recommend tricyclic antidepressants (TCA), selective serotonin reuptake inhibitors (SSRIs) and fluoxetine. But relevant studies show that drug therapy is not ideal (Cox et al., 2012). There is no targeted antidepressant drug for all patients, and drug therapy costs are high and relapse rate is high (Weisz et al., 2017). Some drugs also have side effects such as weight gain, increased blood pressure and impaired sexual function (Pinna, 2015; Cipriani et al., 2016). Fluoxetine has been associated with suicide and high-risk ideational behavior in adolescents (Hammad et al., 2006; Bridge et al., 2007). Cognitive behavioral therapy (CBT) has been confirmed to have moderate to large effect sizes in the treatment of adolescent depression (Klein et al., 2007; Cuijpers et al., 2011). But CBT takes a relatively long time and is expensive (Asarnow et al., 2009). Therefore, it has become the focus of clinicians and researchers to explore the treatment of adolescent depression with convenient operation, low cost and good efficacy.

In recent years, exercise intervention has been recognized as a potentially valuable alternative or adjunct to adolescent depression (Oberste et al., 2018). At present, some meta-analysis has proved that exercise intervention can achieve moderate to large effect size in the treatment of depression (Hu et al., 2020). However, there are many problems with the study results of exercise intervention in adolescent depression. First of all, existing meta-analyses on the efficacy of exercise intervention in the treatment of adolescent depression are controversial. Meta-analysis of Wunram et al. (2018) showed that exercise intervention had a moderate effect size on the treatment of adolescent depression. However, a meta-analysis conducted by Radovic et al. (2017) revealed that exercise intervention had only a small effect size on the treatment of adolescent depression. The controversy of meta-analysis results has brought some confusion to researchers and clinicians, and affected the practical application of exercise intervention in the treatment of adolescent depression. Therefore, it is urgent to review high-quality RCTs to determine the efficacy of exercise on adolescent depression. In the second place, the existing meta-analysis of potential regulatory variables affecting exercise intervention in adolescent depression is not sufficient. And there is debate about the characteristics of the exercise intervention strategies for adolescent depression. Meta-analysis of Wegner et al. (2020) investigated the moderating effects of intervention duration, frequency and type on exercise treatment of adolescent depression. However, the moderating effects of such variables as organization form, single session duration and intensity were not further explored. In addition, there is a lack of retrospective analysis of the sustainability of effects after exercise intervention for adolescent depression.

Therefore, the purpose of this systematic review and meta-analysis is going to investigate the effect size of exercise intervention, and to analyze different exercise intervention strategies for adolescent depression through a meta-analysis of Randomized Controlled Trials. Additionally, we also are going to investigate the sustainability of effects after exercise intervention for adolescent depression, and examine the certainty of evidence in this meta-analysis by using the GRADE framework.

## Methods

The specific operation and writing process of this study followed the PRISMA 2020 guidelines and statement (Page et al., 2021; Supplementary Table 1). The protocol of this systematic review was registered in PROSPERO (CRD42022321683).

### Eligibility criteria

The eligibility criteria of this article were followed the PICOS framework (Liberati et al., 2009). Only the study that conform to the PICOS framework were considered for inclusion. In addition, included studies must be published in English or Chinese in peer-reviewed journals. The eligibility criteria of this review was set before performing a literature search. The eligibility criteria were determined on 1 March 2022, and the last search date was 30 April 2022.

#### Population

Studies were eligible for this review if the participants with a mean age of 10 to 18 years. Furthermore, it is required that the baseline level of depression of the included participants must reach the minimum threshold of depression prescribed by clinical diagnosis or self-rating scale, without other comorbidities (e.g., obesity, cancer, and diabetes).

#### Intervention

Studies were eligible for this review if the treatment meets the American Academy of Sports Medicine’s definition of physical activity. The American Academy of Sports Medicine defines “physical activity” as: “[…] any bodily movement produced by the skeletal muscles that results in energy expenditure above resting levels” (American College of Sports Medicine, 2017). Physical activity, just like The American College of Sports Medicine definition, is an umbrella term that includes subcategories such as sports, leisure activities, and exercise. Exercise, in this article, is defined as a training physical activity intervention that is planned and structured, repetitive and purposeful, leading to a change in fitness (Wegner et al., 2020). Therefore, physical activity includes exercise, but not all physical activity is exercise. Nevertheless, we included physical activity to our search in order to avoid missing some research on exercise.

#### Comparison

Trials were eligible for this review if they compared the effects of exercise and the control group treatment. The control group included in the study requires no additional any exercise or physical activity, which can be educational interventions, recreational games, waiting lists, no interventions, normal medication, etc.

#### Outcome

Included studies were required to report participants’ depressive symptom severity or depression rating Scale scores before and after the trial. Studies use a variety of depression scales like, for example: Depressed Adjective Checklist (DACL), 90-item Self-Rating Depression Scale (SCL-90-R), Achenbach Child Behavior Scale (CBCL), Reynolds Adolescent Depression Scale (RADS), Hamilton self-rating depression scale (Ham-D), Baker self-rating depression scale (BDI, BDI-2).

#### Study design

Only Randomized Controlled Trials were eligible for this article. Additionally, included studies were required there was no significant difference in baseline of all indexes between the experimental group and the control group before the test.

### Search strategy

The electronic search strategy for this study was carried out under the guidance of a research librarian with expertise in systematic reviews. The following databases were searched: PubMed, Medline, EBSCO, Web of Science, SPORTDiscus, PsycINFO, ProQuest, CNKI. The filter of electronic database was used. The search was last conducted 30 April 2022.

Two independent members of the review team (CZ and LC) searched using the operators “AND,” “OR” as well as “*” according to the designed retrieval strategy. Search strategies were modified for each database, and MeSH terms were used when applicable. A variety of search terms were as follows: “Depress*,” “affective symptom,” “affective disorder,” “mood disorder,” “child*,” “adolesc*,” “pubert*,” “girl*,” “boy*,” “youth*,” “teen*,” “exercis*,” “sport*,” “physical activity,” “physical exertion,” “physical training,” “physical education,” “running,” “jogging,” “walking,” “bicycling,” “swimming,” “strength training.” In addition, Meta analyses related to this research topic were searched in each database to supplement the missing literature. The detailed search strategy of this meta-analysis is provided in Supplementary Table 2.

### Data extraction

ENDNOTE X9.0 software was used to remove the repeated processing of literature retrieval. Two researchers (CZ and LC) independently completed literature screening and data extraction according to the above reference inclusion and exclusion criteria. Data extraction included reference information (author, publication year, country), Study design, population information (sample size, age range, sex ratio, description), exercise intervention information (type, Organizational form, frequency, duration, intensity), control intervention information, outcome measure and results (depression, adherence, ITT, dropout, adverse events, follow-up report). For those studies that do not meet the inclusion criteria, review, duplicate publication, irrelevant to the research topic, and have low quality evaluation in the retrieval results, it is eliminated. In addition, the full text of the study was not obtained, and the study whose contact author failed to obtain data was also excluded. Cross-check the results of literature screening and data extraction of two researchers. Any differences between the two searchers (CZ and LC) were resolved in consultation with the third author (JS).

### Methodological quality of studies

Two researchers (CZ and LC) independently used The Assessment of Study Quality and Reporting in Exercise (TESTEX) scale to assess the quality and reporting of the include studies. The TESTEX scale is a specifically tool, designed specifically for use in exercise training studies (Smart et al., 2015). The TESTEX scale consists of 12 criterions, with 15 items (each of which is assigned 1 point), for a maximum score of 15 points. There are 5 criterions of the TESTEX scale for study quality: (1) eligibility criteria specified, (2) randomization specified, (3) allocation concealment of all patients at the time of randomization, (4) groups similar at baseline, (5) blinding of assessor (for at least one key outcome). There are 10 criterions of the TESTEX scale for study reporting: (6) outcome measures assessed in 85% of patients (study withdrawals reported, adverse events reported, session attendance reported), (7) intention-to-treat analysis, (8) reporting of between-group statistical comparisons (primary outcome reported, secondary outcome(s) reported), (9) point measures and measures of variability for all reported outcome measures, (10) activity monitoring in control groups, (11) relative exercise intensity remained constant, (12) exercise volume and energy expenditure. Higher scores on the TESTEX scale reflect better research quality and reporting (Smart et al., 2015). Any differences between the two searchers (CZ and LC) were resolved in consultation with the third author (JS).

### Evaluation of the certainty of evidence

Two researchers (CZ and LC) independently used the GRADE framework to evaluate the certainty of evidence of this study. The GRADE framework consists of the following 4 categories: high level of certainty, moderate level of certainty, low level of certainty, very low level of certainty. The shortcomings that downgrade the certainty of evidence are as follows: limitations in study design, inconsistency, indirectness, imprecision, publication bias. In general, the presence of any of the above shortcomings can downgrade the certainty of evidence by one level, and the presence of all the above shortcoming can downgrade the certainty of evidence by up to three levels. The certainty of evidence is upgraded if one or more of the following factors are present: a large effect, potential bias that reduces the intervention effect, a dose-effect gradient (Schünemann et al., 2013). Any differences between the two searchers (CZ and LC) were resolved in consultation with the third author (JS).

### Statistical analyses

RevMan 5.4 software was used for statistical analysis. In all meta-analyses included, this study used the standardized mean difference (SMD = *M*_1_ – *M_2/_SD_*pooled*_*) to analysis effect size, and the 95% CI to show the 95% confidence interval. Statistical significance of this study was set at *p* ≤ 0.05. The SMD were interpreted with threshold values as follow: 0.2, 0.5 and 0.8 were interpreted as small, medium and large effect sizes, respectively (Cohen, 2013; Oberste et al., 2020). *I*^2^ was used to test the heterogeneity of the included studies, in which 25, 50 and 75% of the *I*^2^ value were the judgment thresholds of low, medium and high heterogeneity, respectively (Higgins et al., 2003). The random effects model was used for all analyses in this study to reduce the bias stemming from the potential heterogeneity between studies (Carter et al., 2016). Based on subgroups analyses, we also examined the moderating potential of antidepressant variables (e.g., organization form, exercise frequency, exercise intensity, exercise type, exercise duration and single exercise session duration) in exercise interventions.

*I*^2^ and *p-*value was used to test the moderating effect (Oberste et al., 2020). Furthermore, if a study included more than one intervention sector, it was analyzed separately in the analysis.

## Results

### Selected studies

**FIGURE 1:**
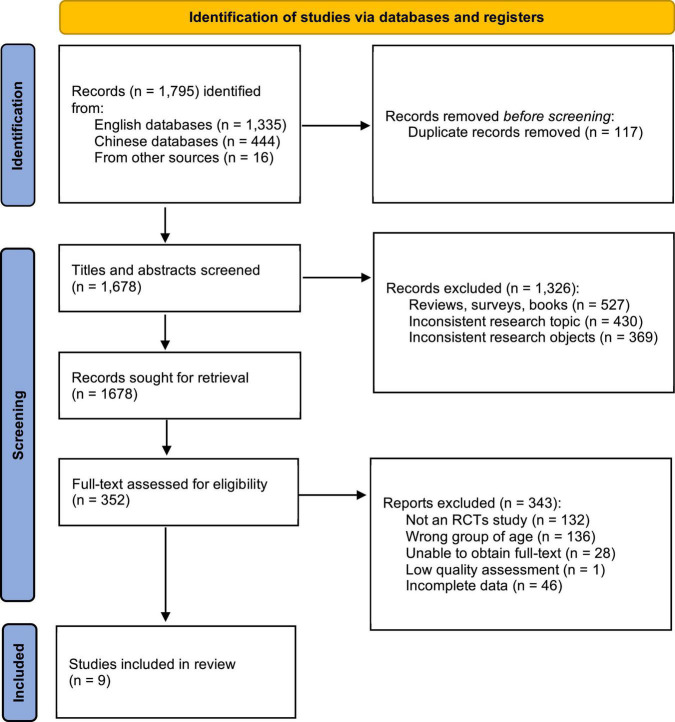
Flow chart of the selection process.

In this study, 1,795 studies were preliminarily retrieved from various electronic databases (1,335 in English databases, 444 in Chinese databases, and 16 obtained from other sources) through the designed retrieval strategy. A total of 117 studies were excluded because they were duplicated. Then, 1,326 unrelated studies were further excluded by reading the title and abstract of the article. After that, on the basis of obtaining, reading and evaluating the full papers, 343 trials were further excluded. In the end, nine studies were included in this systematic review and meta-analysis. Figure 1 shows the more information on the selection process.

### Study characteristics

Table 1 presents the general characteristics information of the studies that were extracted by two reviewers (CZ and LC) using a data extraction form and were summarized.

**TABLE 1:** Characteristics of trials included in the qualitative synthesis of this review.

References & and Country	Study design	Population	Exercise treatment	Comparison intervention	Outcome measure & and results
		1. # of subjects: enrolled (*N*), exercise group (*N*_E_), & and comparison group (*N*c) 2. Age range 3.% of males 4. Description	1. Type of exercise 2. Organizational form 3. Frequency & and duration 4. Intervention intensity		1. Depression 2. Adherence 3. ITT 4. Dropout 5. Adverse events 6. Follow-up report
Burrus (1984)	RCTs	1. *N* = = 45, *N*_E_ = = 30, *N*c = = 15	T1 group:	Education intervention	1. Self-rating scale: DACL
USA		2. 15—18 years-old	1. Mixed exercise		2. 100%
		3. 60%	2. Group training		3. NR
		4. Student	3. 45 min/time, 2 times/week, 9 weeks		4. None out of 45
			4. Moderate		5. NR
			T2 group:		6. NR
			1. Aerobic exercise		
			2. Individual exercise		
			3. 45 min/time, 2 times/week, 9 weeks		
			4. Moderate		
Kanner (1990)	RCTs	1. *N* = = 45, *N*_E_ = = 37, *N*c = = 16	T1 group:	Board games and pool	1. Clinical interviews: CDI-2
USA		2. 11—16 years-old	1. Aerobic exercise	while supervised	2. 27%
		3. 62%	2. Individual exercise		3. NR
		4. Clinical patients	3. 60 min/time, 2 times/week, 6 weeks		4. 12 out of 45
			4. Moderate		5. NR
			T2 group:		6. NR
			1. Aerobic exercise		
			2. Individual exercise		
			3. 60 min/time, 2 times/week, 6 weeks		
			4. Low		
Beffert (1993)	RCTs	1. *N* = = 26, *N*_E_ = = 15, *N*c = = 11	1. Aerobic exercise	Waiting list	1. Clinical interviews: RADS
USA		2. 12—15 years-old	2. Individual exercise		2. 100%
		3. 77%	3. 20 min/time, 2 times/week, 6 weeks		3. NR
		4. Clinical patients	4. Moderate		4. None out of 26
					5. NR
					6. 2 months Follow-up
Jeong et al. (2005)	RCTs	1. *N* = = 40, *N*_E_ = = 20, *N*c = = 20	1. Aerobic exercise	No treatment	1. Self-rating scale: SCL-90-R
South Korea		2. 14—18 years-old	2. Group training		2. 100%
		3. 100%	3. 45 min/time, 3 times/week, 12 weeks		3. Yes
		4. Student	4. Low		4. None out of 40
					5. Yes
					6. NR
Dabidy Roshan et al. (2011)	RCTs	1. *N* = = 24, *N*_E_ = = 12, *N*c = = 12	1. Mixed exercise e	No treatment	1. Observer-rating scale: Ham-D
Iran		2. 15—18 years-old	2. Group training		2. 100%
		3. 100%	3. 50 min/time, 3 times/week, 6 weeks		3. NR
		4. Student	4. Moderate		4. None out of 24
					5. NR
					6. NR
Hughes et al. (2013)	RCTs	1. *N* = = 26, *N*_E_ = = 14, *N*c = = 12	1. Mixed exercise	No treatment	1. Clinical interviews: CDI-2
USA		2. 14—18 years-old	2. Individual exercise		2. 87%
		3. 58%	3. 35 min/time, 3 times/week, 12 weeks		3. NR
		4. Clinical patients	4. Moderate		4. 4 out of 30
					5. Yes
					6. 6—12 months Follow-up
Carter et al. (2015)	RCTs	1. *N* = = 64, *N*_E_ = = 36, *N*c = = 28	1. Mixed exercise	Drug treatment	1. Clinical interviews: CDI-2
Britain		2. 13—17 years-old	2. Individual exercise		2. 75%
		3. 78%	3. 60 min/time, 2 times/week, 6 weeks		3. NR
		4. Clinical patients	4. Low		4. 22 out of 87
					5. Yes
					6. 6 months Follow-up
Liu (2018)	RCTs	1. *N* = = 64, *N*_E_ = = 48, *N*c = = 16	T1 group:	No treatment	1. Clinical interviews: CDI-2
China		2. 10—13 years-old	1. Mixed exercise		2. 100%
		3. 52%	2. Group training		3. NR
		4. Student	3. 30 min/time, 2 times/week, 18 weeks		4. NR
			4. Low		5. NR
			T2 group:		6. NR
			1. Mixed exercise		
			2. Group training		
			3. 45 min/time, 3 times/week, 18 weeks		
			4. Moderate		
			T3 group:		
			1. Mixed exercise		
			2. Group training		
			3. 55 min/time, 3 times/week, 18 weeks		
			4. Vigorous		
Lu et al. (2017)	RCTs	1. *N* = = 91, *N*_E_ = = 46, *N*c = = 45	1. Aerobic exercise	No treatment	1. Self-rating scale: CBCL
China		2. 10—13 years-old	2. Group training		2. 100%
		3. 47%	3. 60 min/time, 4 times/week, 8 weeks		3. NR
		4. Student	4. Low		4. NR
					5. NR
					6. NR

RCTs, randomized controlled trial; T1 group, the experimental group T1 in the study; T2 group, the experimental group T2 in the study; T3 group, the experimental group T3 in the study; DACL, Depression Adjective Checklist; CDI-2, Children’s Depression Inventory, Second Edition; RADS, Reynolds Adolescent Depression Scale; SCL-90-R, Depression scale of the Symptom Checklist-90-Revised; Ham-D, Hamilton Rating Scale for Depression; CBCL, Child Behavior Checklist: CDI, Children’s Depression Inventory.

Nine included studies, all RCTs, published between 1984 and 2018. Only two of the nine studies were reported in the Chinese language (Lu et al., 2017; Liu, 2018), and seven other studies were reported in the English language (Burrus, 1984; Kanner, 1990; Beffert, 1993; Jeong et al., 2005; Dabidy Roshan et al., 2011; Hughes et al., 2013; Carter et al., 2015).

A total of 433 adolescents with depression were included from 9 studies. The sample sizes included in the study ranged from 24 to 91. Only two of the nine studies recruited only female participants (Jeong et al., 2005; Dabidy Roshan et al., 2011), and seven other studies involved both male and females (Burrus, 1984; Kanner, 1990; Beffert, 1993; Hughes et al., 2013; Carter et al., 2015; Lu et al., 2017; Liu, 2018). In addition, the average age of the study participants ranged from 10 to 18 years old. In four of the nine studies, participants were derived from clinical patients who received psychotherapy or medication or control in addition to exercise (Kanner, 1990; Beffert, 1993; Hughes et al., 2013; Carter et al., 2015). In the remaining five studies, participants were in a school setting and received physical activity to treat their depression (Burrus, 1984; Jeong et al., 2005; Dabidy Roshan et al., 2011; Lu et al., 2017; Liu, 2018).

The composition of exercise interventions varies widely across studies. On the type of exercise intervention, six studies used aerobic exercise (Burrus, 1984; Kanner, 1990; Beffert, 1993; Jeong et al., 2005; Dabidy Roshan et al., 2011; Lu et al., 2017), and four studies used mixed exercise (Burrus, 1984; Hughes et al., 2013; Liu, 2018). Mixed exercise, the concept in this study, is defined as regular sessions of two or more types of exercise including aerobic, strengthening, or flexibility exercise (Bidonde et al., 2019).

In terms of organizational form, there were five studies on group training (Burrus, 1984; Jeong et al., 2005; Dabidy Roshan et al., 2011; Lu et al., 2017; Liu, 2018), and five studies on individual sports (Burrus, 1984; Kanner, 1990; Beffert, 1993; Hughes et al., 2013; Carter et al., 2015). The range of exercise length and frequency was included in nine studies were as follows: between 20 and 70 min per session; two to four sessions per week; from 6 to 18 weeks. In terms of exercise intensity, only one study involved high-intensity exercise (Liu, 2018), while the other eight studies were low-intensity or moderate-intensity.

### Methodological quality of included studies

The results of assessment of the two researchers (CZ and LC) were in good agreement (Kappa = 0.87, 95% CI: 0.69 to 0.94, *p* < 0.01). According to the TESTEX criteria, the total TESTEX score, study quality score, and study reporting score of the included studies were 10.00 ± 1.91 (range: 9 to 14, median = 9), 2.62 ± 0.87 (range: 2 to 4, median = 2), 7.46 ± 1.13 (range: 6 to 10, median = 7), respectively. In terms of study quality, the most common concerns were the lack of blinding of assessor (100% of 13 RCTs), the lack of randomization specified (69%), and the lack of allocation concealment of all patients at the time of randomization (69%). In terms of study reporting, the most common concerns were the lack of activity monitoring in control groups (85%), the lack of intention-to-treat analysis (85%), and the lack of adverse events reported (69%). All the included studies for this study obtained a score ≥9 points, so no studies were excluded based on the quality of the study methodology (Thapa et al., 2021). Supplementary Table 3 shows the detail of the TESTEX scores for included studies in this study.

### Results of primary meta-analysis

**FIGURE 2:**
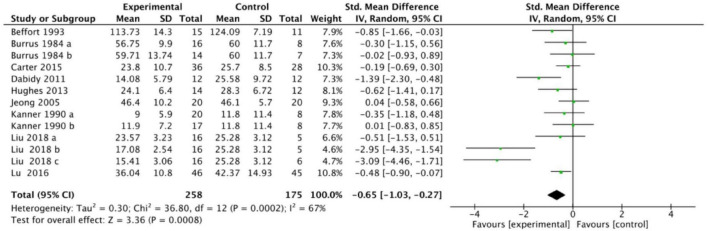
Forest plot of studies included in the primary meta-analysis.

Figure 2 shows the heterogeneity test results of thirteen RCTs included in nine studies. The random effects model was used for to calculate the effect sizes in this study. Three of the nine included studies utilized Multiple arm test. When the study had one or more common intervention groups, in order to avoid the analysis unit error, we divided the common group into two or more sample groups and included two or more (reasonably independent) comparisons (Higgins and Green, 2011). After pooling effect size estimates, the overall combined effect size was SMD = −0.65, 95% CI: −1.03 to −0.27. The result was statistical significance (*p* < 0.01). This study Heterogeneity was substantial (*T*^2^ = 0.30, *I*^2^ = 67%, *p* < 0.01). The result indicate that exercise intervention has a therapeutic effect on adolescent depression, reaching a moderate-to-large effect size.

### Moderator analysis

This study Heterogeneity was substantial (*T*^2^ = 0.30, *I*^2^ = 67%, *p* < 0.01). The moderating effects were described by subgroup analysis of the exercise variables and methodological features of included studies. A summary of our moderator analysis is presented in Table 2.

**TABLE 2:** Moderator analysis of this review.

Variable	Subgroups	*K*	*n*	SMD value (95% CI)	Two-sided Hypotheses		Heterogeneity test between groups		
					*Z*	*p*	χ^2^	*p*	*I*^2^ (%)
Exercise type	Aerobic exercise	6	231	−0.32 (−0.59, −0.05)	2.35	<0.05	2.31	<0.05	76.1%
	Mixed exercise	7	202	−1.14 (−1.88, −0.40)	3.03	<0.01			
Organizational form	individual exercise	6	190	−0.32 (−0.61, −0.02)	2.08	<0.05	3.33	<0.05	72.4%
	Group training	7	243	−1.06 (−1.77, 0.35)	2.87	<0.01			
Intervention duration	<8 weeks	5	167	−0.50 (−0.95, −0.04)	2.15	<0.05	2.34	0.31	14.7%
	8∼12 weeks	3	136	−0.39 (−0.73, −0.04)	2.18	<0.05			
	>12 weeks	5	130	−1.30 (−2.42, −0.18)	2.27	<0.05			
Single session duration	20 to 60 min/time	9	225	−0.94 (−1.55, −0.33)	3.01	<0.01	3.27	<0.05	69.4%
	≥60 min/time	4	208	−0.32 (−0.60, −0.04)	2.23	<0.05			
Intervention frequency	1∼2 times/week	7	209	−0.29 (−0.58, −0.03)	1.96	<0.05	4.64	<0.05	78.5%
	≥3 times/week	6	224	−1.22 (−2.01, −0.42)	3.01	<0.01			
Intervention intensity	Low	5	241	−0.27 (−0.53, −0.01)	2.01	<0.05	4.92	<0.05	79.7%
	Moderate to vigorous	8	192	−1.06 (−1.71, −0.41)	3.19	<0.01			
Context of participant	School students	9	290	−0.89 (−1.45, −0.33)	3.13	<0.01	3.53	<0.05	71.7%
	Clinical patients	4	143	−0.27 (−0.61, −0.13)	2.21	<0.05			

k, number of effect size estimates; N, number of participants; SMD, standardized mean different; CI, confidence interval.

### Variables of exercise intervention

#### Type of exercise

Subgroup analysis revealed that there were significant differences in the therapeutic effect of different exercise types on adolescent depression (χ^2^ = 2.31, *p* < 0.05, *I^2^* = 76.1%). The data indicate that exercise type influences the relationship between exercise intervention and treatment for depression in adolescents. Mixed exercise had a large effect size (SMD = −1.14, 95% CI: −1.88 to −0.40, *p* < 0.01), and aerobic exercise had a medium effect size (SMD = −0.32, 95% CI: −0.59 to −0.05, *p* < 0.05).

#### Organizational form

Subgroup analysis showed that there were significant differences in the therapeutic effect of different tissue forms on adolescent depression (χ^2^ = 3.33, *p* < 0.05, *I^2^* = 72.4%). The data suggest that organizational form influences the relationship between exercise intervention and treatment for adolescent depression. Group training had a large effect size (SMD = −1.06, 95% CI: −1.77 to −0.35, *p* < 0.01) on the treatment of adolescent depression, while individual exercise had a medium effect size (SMD = −0.32, 95% CI: −0.61 to −0.02, *p* < 0.05).

#### Intervention duration

Subgroup analysis revealed a small-to-moderate effect size when intervention duration was less than 8 weeks (SMD = −0.50, 95% CI: −0.90 to −0.04, *p* < 0.05). When the intervention lasted 8 to 12 weeks, a small-to-moderate effect size was achieved (SMD = −0.39, 95% CI: −0.73 to −0.04, *p* < 0.05). When the intervention lasted more than 12 weeks, the large effect size was achieved (SMD = −1.30, 95% CI: −2.42 to −0.18, *p* < 0.05). However, the difference between the effect sizes of subgroups did not reach statistical significance (χ^2^ = 2.34, *p* = 0.31, *I^2^* = 14.7%).

#### Single session duration

Subgroup analysis revealed that there were significant differences in the therapeutic effect of different single session duration on adolescent depression (χ^2^ = 3.27, *p* < 0.05, *I^2^* = 69.4%). The data suggest that the single session duration affects the relationship between exercise intervention and treatment for depression in adolescents. When single session duration was 20 to 60 min/time, a moderate-to-large effect size was achieved (SMD = −0.94, 95% CI: −1.55 to −0.33, *p* < 0.01). When the single session duration greater than or equal to 60 min/time, a small-to-moderate effect size was achieved (SMD = −0.32, 95% CI: −0.60 to −0.04, *p* < 0.05).

#### Intervention frequency

Subgroup analysis revealed that there were significant differences in the therapeutic effect of different intervention frequency on adolescent depression (χ^2^ = 4.64, *p* < 0.05, *I^2^* = 78.5%). The data suggest that the intervention frequency affects the relationship between exercise intervention and treatment for depression in adolescents. When the intervention frequency was 1 to 2 times/week, a small-to-moderate effect size was achieved (SMD = −0.29, 95% CI: −0.58 to −0.03, *p* < 0.05). When the intervention frequency was 3 times/week or more, the effect size was large (SMD = −1.22, 95% CI: −2.20 to −0.42, *p* < 0.01).

#### Intervention intensity

Subgroup analysis revealed that there were significant differences in the therapeutic effects of different intervention intensities on adolescent depression (χ^2^ = 4.92, *p* < 0.05, *I^2^* = 79.7%). The results showed that intervention intensity had an effect on the relationship between exercise intervention and treatment of adolescent depression. Low intensity exercise had a small-to-moderate effect size on the treatment of depression in adolescents (SMD = −0.27, 95% CI: −0.53 to −0.01, *p* < 0.05). Moderate to high intensity exercise achieved large effect size (SMD = −1.06, 95% CI: −1.71 to −0.41, *p* < 0.01).

### Methodological features of included studies

We compared the antidepressant effects of exercise on adolescent depression in different contexts of participant. Subgroup analysis revealed that there were significant differences in the therapeutic effects of different contexts on adolescent depression (χ^2^ = 3.52, *p <* 0.05, *I*^2^ = 71.7%). The results showed that intervention intensity had an effect on the relationship between exercise intervention and treatment of adolescent depression. Exercise intervention has a moderate-to-large effect size on school students (SMD = −0.89, 95% CI: −1.45 to −0.33, *p <* 0.01). Exercise intervention has a small to moderate effect size on clinical patients (SMD = −0.27, 95% CI: −0.61 to −0.13, *p <* 0.01).

### Analysis of the sustainability of the effects after exercise intervention termination

Table 3 shows follow-up data from the three included studies that reported the sustainability of effects after exercise treatment termination. We chose to describe the results of the three studies that reported the follow-up data due to too low statistical test power. Two of there studies reported specific data of sustained relief of depressive symptoms after the exercise intervention (Beffert, 1993; Carter et al., 2015). One study reported the remission rate of depressive symptoms after the exercise intervention (Hughes et al., 2013), Beffert (1993) reported that the antidepressant effects of exercise intervention still remained 5 months after the exercise intervention ended (SMD = −0.94, 95% CI: −1.78 to −0.11). In the study by Carter et al. (2015), at 6 months after the end of exercise intervention, the antidepressant effects of exercise intervention approached almost a moderate level (SMD = 0.39, 95% CI = −1.00 to 0.22). At post-intervention, Hughes et al. (2013) reported that the remission for all exercise group participant remained stable until 40 weeks after the end of exercise intervention.

**TABLE 3:** Follow-up reports included in the reference.

References	Follow-up report		
	The immediate effect of the end of the exercise intervention	Length of follow-up	Subsequent effect changes
Beffert, 1993	SMD = −0.85, 95% CI: −1.66∼−0.03	2 months	SMD = −0.94, 95% CI: −1.78∼ −0.11
Carter et al., 2015	SMD = −0.19, 95% CI: −0.69∼ 0.30	6 months	SMD = −0.39, 95% CI: −1.00∼ 0.22
Hughes et al., 2013	SMD = −0.62, 95% CI: −1.41∼ 0.17	6–12 months	1. After 14 weeks, 86% of the trial group no longer had clinical symptoms of depression. 2. After 40 weeks, no clinical symptoms of depression were found in all subjects.

### Publication bias analysis

**FIGURE 3:**
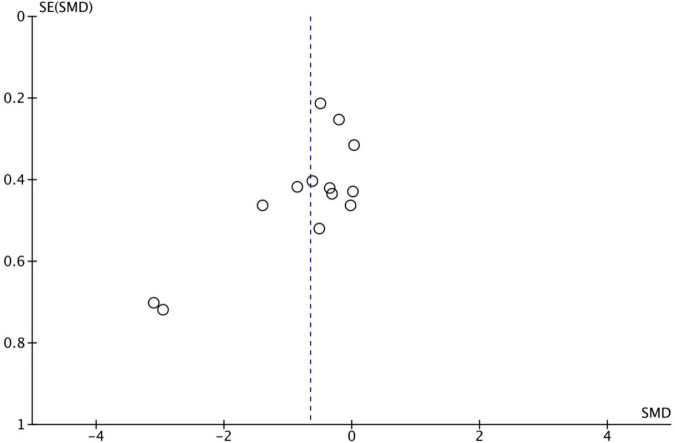
Results of publication bias analysis.

Figure 3 shows the publication bias risk test of the paper included in this study. The risk of publication bias can be tested by funnel plots (Rothstein et al., 2005). The results showed that the included studies were evenly distributed around the combined effect size SMD, and the left and right sides were basically symmetrical. There were two studies with some degree of deviation. It can be seen that there may be some publication bias in the 13 RCTs trials, but it is not very serious. Within the acceptable range, the stability of the results of this meta-analysis will not be seriously affected.

### Certainty of evidence

We used the GRADE framework to evaluate the certainty of evidence of this study. Following the GRADE framework, owing to the included studies contained some methodological limitations, we rated the certainty of evidence the primary meta-analysis as moderate evidence. In terms of “study design,” we identified some limitations, and downgraded one level the certainty of evidence. In terms of “inconsistency,” “indirectness,” “imprecision” or “publication bias,” we did not downgrade the certainty of evidence. Supplementary Tables 4, 5 shows the more information of the GRADE rating.

## Discussion

### Effect of exercise on the treatment of adolescent depression

This study conducted a meta-analysis of thirteen RCTs trials in nine studies, which comprised a total of 433 adolescents. The results showed that the effect of exercise on the treatment of adolescent depression in comparison to control treatments reached moderate pooled effects, with a confidence interval that ranged from a medium to a large effect (SMD = −0.65, 95% CI: −1.03 to −0.27, *p* < 0.01). Our results are consistent with the results of some previous meta-analyses (Brynhildur et al., 2020; Oberste et al., 2020). Moreover, the finding of the antidepressant effect size in our study, is slightly higher than the effect size recommended by clinical guidelines for drug treatment of adolescent depression (SMD = −0.48) (Zhou et al., 2015). It is worth affirming that there were no reports of adverse events caused by exercise intervention in the included studies.

We found that compared with clinically confirmed patients (confirmed by structured clinical interview), exercise intervention had a greater therapeutic effect on school students (identified by the Self-Rating Scale). This result may be related to the bias in the screening process of some subjects and the inclusion of some false positive samples. As we all know, clinical interview is the gold standard for the diagnosis of adolescent depression (Haugen et al., 2016), while but the sensitivity of most self-rating scales for adolescent depression is less than 75% at present (Salle et al., 2012). The difference in sensitivity between the two diagnostic methods means that a higher percentage of false positive samples will be screened out by the self-rating Depression Scale compared to structured clinical interviews. Such misdiagnosis may influence trial results and thus interfere with researchers’ judgment of the anti-depressive effect of exercise intervention (Bailey et al., 2018). Therefore, it is suggested to set up structured clinical interviews for blind diagnosis by clinical experts in future studies to test whether the subjects are eligible for inclusion.

However, owing to the included studies contained some methodological limitations (according to the TESTEX criteria), we rated the certainty of evidence the primary meta-analysis as moderate evidence. Thus, we have a moderate degree of confidence in the estimate of the effect, and the true value is likely to be close to the estimate, but there are still different possibilities. More rigorous studies are still needed to verify the antidepressant effect size on adolescent depression.

### Exercise intervention strategies for adolescent depression

We found that mixed exercise had a large effect size on the treatment of depression in adolescents, and its effect was significantly better than the moderate effect of aerobic exercise. Aerobic exercise is the most commonly used type of exercise clinically recommended for the treatment of depression (Morres et al., 2019), but mixed exercise has significant advantages over aerobic exercise alone (Hishikawa et al., 2019). Some studies have found that when the exercise intervention program is aerobic, anaerobic, resistance, competition and sports games and other mixed exercise, patients with depression symptoms are not only effectively treated, but also have multiple benefits such as physical function improvement, emotional regulation and improvement, and quality of life improvement (Ranjbar et al., 2015). Moreover, the compliance rate of subjects was over 80% (Korman et al., 2020). For adolescents with depression who prefer different forms of stimulation, mixed exercise can stimulate their enthusiasm for exercise participation and treatment compliance rate more than single exercise to some extent.

As for the organizational form, we found that group form exercise achieved a large effect size in the treatment of adolescent depression, and its treatment effect was significantly better than the medium effect size of individual form exercise. Previous studies have shown that compared with the boring individual form of exercise (e.g., running or walking), interpersonal interaction, social support and sports professional guidance in group form of exercise can help patients with depression to relieve depressive symptoms more quickly (Carter et al., 2016; Meyer et al., 2016). Loss of interest in social activities is one of the most important symptoms in depressed individuals (Sani et al., 2014). Group form of exercise can provide adolescents with depression the opportunity to express their thoughts, memories and emotions (Harris and Orth, 2020), promote communication with others, and promote later social integration (Dupuis et al., 2011).

As for the intervention duration, we found that exercise intervention had the best effect on adolescent depression when the intervention was more than 12 weeks, followed by less than 8 weeks, and finally 8 to 12 weeks. Although there was small statistical test power might explain the lack of statistical significance between the effects of the three subgroups, but the result suggested that the therapeutic effect of exercise on adolescent depression may fluctuate to some extent. Previous studies have found that the therapeutic effect of exercise intervention on depression will lag about 3–4 weeks in time (Michele and Giosuè, 2010), and significant improvement can be achieved only after lasting at least 9 weeks (Stanton and Reaburn, 2014). This may be related to such factors as depression patients’ doubt about treatment methods, delay in self-feeling improvement and resistance to receiving improvement effects (Mota-Pereira et al., 2011). Other studies have found that the effect of exercise antidepressant therapy will be flat, that is, with the increase of intervention duration, the effect value will decrease to a certain extent (Schuch et al., 2016). But more research is still needed to confirm this fluctuation phenomenon of exercise treatment effect on adolescent depression.

As for the single session duration, we found that the single session duration of 20 to 60 min/time was significantly better than a single duration of more than 60 min/time. Previous meta-analyses at different periods have also shown that different exercise duration (range: 20 to 60 min/time) has a significant positive effect on the treatment of depression (Martinsen, 2008; Stanton and Reaburn, 2014). Other studies suggest that the antidepressant effect of exercise needs to reach a certain time transition point, among which at least 30 min/time is the general view of exercise antidepressant (Meyer et al., 2016). This phenomenon may be related to the time-dependent response of human neurophysiological processes to exercise (Duclos and Tabarin, 2016). Maladjusted levels of norepinephrine, brain-derived neurotrophic factor (BDNF), and 5-hydroxytryptamine (5-HT) have been shown to be potentially underlying causes of depression (Carek et al., 2011; Xie et al., 2021). Studies have shown that exercise can affect the production of norepinephrine, brain-derived neurotrophic factor (BDNF) and serotonin (5-HT), with concentrations of these neurotransmitters varying at different points in time (Medina et al., 2015; Kandola et al., 2019). However, it is not clear which single exercise duration has the optimal effect on the relevant neurotransmitters.

As for the exercise frequencies, we found that more than 3 times/week is significantly better than 1 to 2 times/week in the treatment of adolescent depression. For the antidepressant effects of different exercise frequencies, previous studies have suggested that high frequency exercise (3 to 5 times/week) is more effective than low frequency exercise (1 time/week) (Legrand and Heuze, 2007). Some studies have suggested that exercise intervention prescription for depression can be limited to 4 to 5 times/week, and for large intensity exercise program, it can be one time/week or 2 to 3 times/week, but the maximum interval between two events should not exceed 3 days, and it is difficult to achieve the ideal effect after the interval exceeds 3 days (Medina et al., 2015).

As for exercise intensity, we found that moderate to vigorous intensity exercise had a large effect on the treatment of adolescent depression, and the treatment effect was significantly better than low intensity exercise. Short-term intermittent aerobic exercise that reaches 80% of maximum heart rate has been shown to effectively reduce depressive symptoms (Herring et al., 2017), and long-term aerobic exercise that reaches 70% to 80% of maximum heart rate has almost the same improvement effect on depression as antidepressants (Shimoda et al., 2017). Some studies have found that low-intensity, medium-intensity and high-intensity exercise both can improve depressive symptoms (Meyer et al., 2016), but in comparison, moderate to vigorous intensity exercise has better antidepressant effects than low intensity exercise (Silveira et al., 2013).

### The sustainability of the effects after exercise intervention termination

We found a follow-up effect of the exercise intervention, which further improved the treatment of adolescent depression after the exercise was stopped. The follow-up report of Beffert (1993) showed that the therapeutic effect of exercise intervention on adolescent depression increased slightly 2 months after the end of exercise, with SMD = −0.85 (95% CI: −1.66 to −0.03) rising to SMD = −0.94 (95% CI: −1.78 to −0.11). The follow-up report of Carter et al. (2015) showed that the therapeutic effect of exercise intervention on adolescent depression increased from small effect size (SMD = −0.19, 95% CI: −0.69 to 0.30) to medium effect size (SMD = −0.39, 95% CI: −1.00 to 0.22). In the follow-up report of Hughes et al. (2013) showed that 86% of the test group no longer showed clinical symptoms of depression 14 weeks after the exercise intervention, and 40 weeks after the end of exercise therapy, the clinical symptoms of depression were effectively alleviated in almost all subjects in the experimental group. However, the duration and internal mechanism of the follow-up effect of exercise intervention on depression remain unclear. Some studies speculated that the continuation of the antidepressant effect of exercise intervention might be related to the change of subjects’ living habits (Hoffman et al., 2011; Helgadottir et al., 2017). Adolescent depression patients had a high compliance with exercise intervention, and the shedding rate of test group and control group was much lower than the data reported by previous psychotherapy (De-Haan et al., 2013) and related medication (Hetrick et al., 2012). To a certain extent, the exercise habits formed by the exercise intervention are beneficial to the maintenance of exercise habits after the exercise intervention, thus reducing their depression state. However, we need to treat this conclusion with caution for the sustainability of effects after exercise treatment termination. High-quality RCTs research, Significantly, is needed to confirm this conclusion.

### Limitations of this meta-analysis

In this systematic review and meta-analysis, we included only articles published in English or Chinese. Therefore, studies in other languages and unpublished have not been included. There may be language bias and scattered bias to some extent. In addition, the strength of our conclusions is limited by the small number of included studies and lack of large-sample and high-quality RCT studies on exercise intervention in the treatment of adolescent depression.

### General practice implications

Currently, exercise is considered by clinicians and the general public as an effective way to prevent or treat depression in adolescents, and this is supported by numerous studies (Carek et al., 2011; Medina et al., 2015; Xie et al., 2021). We suggest that the following points should be considered in the future research or clinical practice of exercise intervention in adolescent depression: First, when exercise is used as monotherapy, adjuvant therapy or combination therapy, the degree of depressive symptoms of participants should be considered. It is worth noting that future studies should consider establishing structured clinical interviews and expert blind diagnosis to reduce false positive samples. Secondly, the exercise plan should be personalized and interesting. In other words, the exercise plan should be negotiated by professionals according to the participants’ own factors (e.g., age, gender, athletic ability, economic ability, time, personal preference). Third, exercise programs should be conducted under the supervision or guidance of a physical therapist, personal trainer or other professional, with follow-up records and feedback.

## Conclusion

This systematic review and meta-analysis systematically reviewed studies that look at exercise intervention with adolescent depression. Our review indicated that exercise intervention can effectively treat adolescent depression with a moderate effect size. Our results recommended that adolescents with depression undertake moderate to high intensity group mixed exercise for more than 12 weeks, 20 to 60 min/time, more than 3 times/week. Additionally, our study also shows that the antidepressant effects remained for a long time after the end of exercise interventions. However, following the GRADE framework, we rated the certainty of evidence the primary meta-analysis as moderate evidence due to some methodological limitations of included studies. Thus, we have a moderate degree of confidence in the estimate of the effect, and the true value is likely to be close to the estimate, but there are still different possibilities. Therefore, more rigorous studies are still needed to verify the antidepressant effect size on adolescent depression.

## Data availability statement

The original contributions presented in this study are included in the article/Supplementary material, further inquiries can be directed to the corresponding author.

## Author contributions

CZ and JS conceptualized and designed the idea of the study. CZ, JS, and LC designed the data collection instruments, collected the data, carried out the initial analyses. CZ and LC drafted the initial manuscript and reviewed and revised the manuscript. XC, YW, and SW reviewed and revised the manuscript. All authors have read and approved the final version of the manuscript and agreed with the order of presentation of the authors.
