# Supplementary material for: The strategies of exercise intervention for adolescent depression: A meta-analysis of randomized controlled trials
Source: Front Psychol. 2023 Jan 4;13:974382. doi: 10.3389/fpsyg.2022.974382 (PMC9846179; doi:10.3389/fpsyg.2022.974382)
Supplement: Supplementary file 2 [file Data_Sheet_2.PDF]

Table S2. Search terms.

**Population**	MeSH search: exp adolescent/ or exp child/students/teen/puberty<br><br>Title search: boy$1 or girl$1 or kid$1 or school$ or preschool$ or juvenil$ or under?age$ or teen$ or minor$ or pubescen$ or young people or young person$ or youth$ or student$).m_titl. child$ or adolescen$ or pediatric$ or teen$ or pubert$).m_titl
**AND**	
**Outcome**	MeSH search: Mental health/mental disorders/ or adjustment disorders/ or affective disorders/ mental disorders/ or dissociative disorders/ or neurotic disorders/ or affective symptom/ or mood disorder<br><br>Title search: sadness or low mood or melanchol* or depress* or dysphor* or dysthymi* or affective symptom or affective disorder or mood disorder
**AND**	
**Intervention**	MeSH search: exp exercise/or physical fitness/ or exp sports/ exp exercise therapy/ muscle stretching exercises/ or physical exertion/ or physical training/ or physical education/ or running/ or jogging/ or walking/ or bicycling/ or swimming/ or strength training<br>Title search: exercis* or physical activit* or sport* or athletic* or physical train* or physical educat*).m_titl.
**Study design limits**	Above search limited to: English language and Chinese language, only human trials (clinical trial all, or clinical trial or comparative study or controlled clinical trial or pragmatic clinical trial or randomized controlled trial)
**Note:** MeSH = Medical Subject Heading	
