# Supplementary material for: The strategies of exercise intervention for adolescent depression: A meta-analysis of randomized controlled trials
Source: Front Psychol. 2023 Jan 4;13:974382. doi: 10.3389/fpsyg.2022.974382 (PMC9846179; doi:10.3389/fpsyg.2022.974382)
Supplement: Supplementary file 3 [file Data_Sheet_3.PDF]

TABLE S3 | Methodological quality score of the included studies in this study.

References	Items					Points of study quality	Items							Points of Study reporting	Total points
	1	2	3	4	5		6	7	8	9	10	11	12		
Burrus 1984(a)	1	0	0	1	0	2	2	0	2	1	0	1	1	7	9
Burrus 1984(b)	1	0	0	1	0	2	2	0	2	1	0	1	1	7	9
Kanner 1990(a)	1	0	0	1	0	2	2	0	2	1	0	1	1	7	9
Kanner 1990(b)	1	0	0	1	0	2	2	0	2	1	0	1	1	7	9
Beffert 1993	1	1	0	1	0	3	2	0	2	1	0	1	1	7	9
Jeong 2005	1	1	1	1	0	4	3	1	2	1	0	1	1	9	13
Dabidy 2011	1	0	1	1	0	3	1	0	2	1	0	1	1	6	9
Hughes 2013	1	1	1	1	0	4	3	0	2	1	1	1	1	9	13
Carter 2015	1	1	1	1	0	4	3	1	2	1	1	1	1	10	14
Liu 2018(a)	1	0	0	1	0	2	2	0	2	1	0	1	1	7	9
Liu 2018(b)	1	0	0	1	0	2	2	0	2	1	0	1	1	7	9
Liu 2018(c)	1	0	0	1	0	2	2	0	2	1	0	1	1	7	9
Lu 2016	1	0	0	1	0	2	2	0	2	1	0	1	1	7	9

Item 1, eligibility criteria specified; Item 2, randomization specified; Item 3, allocation concealment of all patients at the time of randomization; Item 4, groups similar at baseline; Item 5, blinding of assessor (for at least one key outcome); Item 6, outcome measures assessed in 85% of patients (study withdrawals reported, adverse events reported, session attendance reported); Item 7, intention-to-treat analysis; Item 8, reporting of between-group statistical comparisons (primary outcome reported, secondary outcome(s) reported); Item 9, point measures and measures of variability for all reported outcome measures; Item 10, activity monitoring in control groups; Item 11, relative exercise intensity remained constant; Item 12, exercise volume and energy expenditure.
