# Supplementary material for: The strategies of exercise intervention for adolescent depression: A meta-analysis of randomized controlled trials
Source: Front Psychol. 2023 Jan 4;13:974382. doi: 10.3389/fpsyg.2022.974382 (PMC9846179; doi:10.3389/fpsyg.2022.974382)
Supplement: Supplementary file 4 [file Data_Sheet_4.PDF]

TABLE S4 | GRADE rating assessing the certainty of the evidence that contributed to the summary effect of exercise intervention on depressive symptoms calculated in the primary meta-analysis.

RCT level evidence: HIGH ⊕⊕⊕⊕		
**1. Limitation in study design (risk of bias):** some limitations	**Action:** downgrading one levels ⊖	**Reason:** We included RCTs into this review, according to the TESTEX criteria, but with some limitations. In terms of study qualit the 13 RCTs, the most common concerns were the lack of blinding of assessor (100% of 13 RCTs), the lack of randomization speci (69%), and the lack of allocation concealment of all patients at the time of randomization (69%). Moreover, it is difficult to ach double blindness in exercise intervention, so it is considered that the blind method design in this study is not the source of limitation terms of study reporting, the most common concerns were the lack of activity monitoring in control groups (85%), the lack of intent to-treat analysis (85%), and the lack of adverse events reported (69%).<br>Therefore, we downgraded the certainty of the evidence by one levels due to these study limitations.
**2. Inconsistency:** Unlikely	**Action:** do not downgrade	**Reasons:** We have searched the RCTs that fit the criteria. We have compared the effects of exercise intervention and control group adolescent depression. 95% CI of all trials included into our study overlapped.<br>Therefore, we did not downgrade the certainty of evidence.
**3. Indirectness:** Unlikely	**Action:** do not downgrade	**Reason:** All included trials were relevant to the review question, no indirect comparators were used, all trials recruited adolescents with at least threshold levels of depressive symptoms, all reported depression symptoms as outcomes.<br>Therefore, we did not downgrade the certainty of evidence
**4. Imprecision:** Unlikely	**Action:** do not downgrade	**Reason:** The total number of participants included into the meta-analysis was 433. That is more than the number of patients genera by a conventional sample size calculation for a single adequately powered trial. The effect size is larger than 0.2 standard deviation 95% CI of summary effect does not cross the line of no effect. 95% CI of summary effect is relatively narrow with a lower level above .2 standard deviations.<br>Therefore, we did not downgrade the certainty of evidence.
**5. Publication Bias**: Unlikely	**Action:** do not downgrade	**Reason:** There were two studies with some degree of deviation. It can be seen that there may be some publication bias in the 13 R trials, but it is not very serious. Within the acceptable range, the stability of the results of this meta-analysis will not be seriously affec<br>Therefore, we did not downgrade the certainty of evidence.
**Overall certainty of evidence rating:** Moderate ⊕⊕⊕⊖<br><br>**Interpretation**<br><br>Moderate ⊕⊕⊕⊖ — Further research is likely to have an important impact on our confidence in the estimate of effect and may change the estimate.		
