# Supplementary material for: The strategies of exercise intervention for adolescent depression: A meta-analysis of randomized controlled trials
Source: Front Psychol. 2023 Jan 4;13:974382. doi: 10.3389/fpsyg.2022.974382 (PMC9846179; doi:10.3389/fpsyg.2022.974382)
Supplement: Supplementary file 5 [file Data_Sheet_5.PDF]

TABLE S5 | Summary of Findings for the Main Outcome.

Exercise Intervention compared to control for adolescents suffering from depression
Population: adolescents with depression
Setting: any setting
Intervention: exercise intervention
Comparison: no additional any exercise or physical activity

**Outcomes**	**No of Participants (studies, effect sizes estimates)**	**Quality of the evidence (GRADE)**	**Illustrative comparative risks (95% CI)**		**Comments**
			**Assumed risk**	**Corresponding risk**	
			**Control**	**Exercise**	
**Symptoms of depression**<br>Different scales<br>Post intervention	433<br>(9 studies, 13 RCTs)	Moderate<br>⊕⊕⊕⊖[1,2,3]	The mean symptoms of depression in the control group was **0**	The mean symptoms of depression in the intervention group was **.65 standard deviations lower** (1.03 to .27 lower)	SMD -.65 (95% CI: -.03 to - .27).<br><br>The effect sizes is interpreted as 'moderate' (using Cohen's rule of thumb)

*__The risk in the intervention group__ (and its 95% CI) is based on the assumed risk in the comparison group and the **relative effect** of the intervention (and its 95% CI).
CI: Confidence interval

GRADE Working Group grades of evidence
High quality: Further research is very unlikely to change our confidence in the estimate of effect.
Moderate quality: Further research is likely to have an important impact on our confidence in the estimate of effect and may change the estimate.
Low quality: Further research is very likely to have an important impact on our confidence in the estimate of effect and is likely to change the estimate.
Very low quality: We are very uncertain about the estimate.

[1] We included RCTs into this review, according to the TESTEX criteria, but with some limitations.
[2] In terms of study quality of the 13 RCTs, the most common concerns were the lack of blinding of assessor (100% of 13 RCTs), the lack of randomization specified (69%), and the lack of allocation concealment of all patients at the time of randomization (69%). Moreover, it is difficult to achieve double blindness in exercise intervention, so it is considered that the blind method design in this study is not the source of limitations.
[3] In terms of study reporting, the most common concerns were the lack of activity monitoring in control groups (85%), the lack of intention-to-treat analysis (85%), and the lack of adverse events reported (69%).
